# Supplementary material for: Spectral field mapping in plasmonic nanostructures with nanometer resolution
Source: Nat Commun. 2018 Oct 11;9:4207. doi: 10.1038/s41467-018-06572-9 (PMC6181996; doi:10.1038/s41467-018-06572-9)
Supplement: Supplementary file 1 — Supplementary Information [file 41467_2018_6572_MOESM1_ESM.pdf]

Supplementary Information: Spectral Field Mapping in Plasmonic Nanostructures with Nanometer Resolution,[1] J. Krehl,[1] G. Guzzinati,[2] J. Schultz,[1] P. Potapov,[1] D. Pohl,[1, 3] Jérôme Martin,[4] J. Verbeeck,[2] A. Fery,[5] B. Büchner,[1] and A. Lubk[1]

[1] *IFW Dresden, Helmholtzstr. 20, 01069 Dresden, Germany*
[2] *EMAT, University of Antwerp, Groenenborgerlaan 171, 2020 Antwerp, Belgium*
[3] *Dresden Center for Nanoanalysis, TU Dresden, 01062 Dresden, Germany.*
[4] *Institut Charles Delaunay – Laboratoire de nanotechnologies et d'instrumentation optique, UMR CNRS 6281, Université de Technologie de Troyes, Troyes 10010, France*
[5] *IPF Dresden, Hohe Str. 3, 01069 Dresden, Germany*

Supplementary Note 1. Quantum Formalism

The following lines describe the total information content, which may be retrieved from the full (in)elastic EFDP maps. We begin with a short compilation of the original work of Rodenburg [5], who established the phase space (Wigner function) formulation of ptychography, i.e., the reconstruction method applied to the EFDP maps. Under axial scattering conditions the density matrix of the final state (i.e., the matrix elements of the density operator in spatial representation)

$$\rho_f\left(\mathbf{r},\mathbf{r}',\omega\right) = \left\langle \mathbf{r}\left|\hat{\rho}_f\right|\mathbf{r}'\right\rangle$$

is given by a simple product of the incoming state ρ_i and the so-called mutual object transparency [2]

$$\rho_f\left(\mathbf{r},\mathbf{r}',\omega\right) = T\left(\mathbf{r},\mathbf{r}',\omega\right)\rho_i\left(\mathbf{r},\mathbf{r}'\right) . \tag{1}$$

This expression is a generalization of the more familiar product of the initial wave function Ψ_i with a transmission function

$$\Psi_f\left(\mathbf{r}\right) = T\left(\mathbf{r}\right)\Psi_i\left(\mathbf{r}\right) , \tag{2}$$

valid for axial elastic scattering. Now, we write the far field intensity recorded in the diffraction pattern for a particular probe position $\mathbf{r}_i$ in the axial approximation

$$\begin{aligned} I\left(\mathbf{k}\right) &= \int \mathrm{d}^2r\mathrm{d}^2r' \left\langle \mathbf{k}\left|\mathbf{r}\right.\right\rangle\left\langle \mathbf{r}\right|\hat{\rho}_f\left|\mathbf{r}'\right\rangle\left\langle \mathbf{r}'\right|\left.\mathbf{k}\right\rangle \\ &= \frac{1}{2\pi}\int \mathrm{d}^2r\mathrm{d}^2r''\, e^{-i\mathbf{kr}}T\left(\mathbf{r},\mathbf{r}'\right)\rho_i\left(\mathbf{r}-\mathbf{r}_i,\mathbf{r}'-\mathbf{r}_i\right)e^{i\mathbf{kr}'} . \end{aligned} \tag{3}$$

Inserting the Wigner transform of the mutual object transparency

$$T_W(\mathbf{r},\mathbf{k}) = \int T\left(\boldsymbol{r}+\frac{1}{2}\boldsymbol{r}',\boldsymbol{r}-\frac{1}{2}\boldsymbol{r}'\right)e^{-i\mathbf{kr}'}\mathrm{d}^2r' \tag{4}$$

and the probe state

$$W_i\left(\mathbf{r},\mathbf{k}\right) = \frac{1}{2\pi}\int \rho_i\left(\boldsymbol{r}+\frac{1}{2}\boldsymbol{r}',\boldsymbol{r}-\frac{1}{2}\boldsymbol{r}'\right)e^{-i\mathbf{kr}'}\mathrm{d}^2r' \tag{5}$$

into (3) one obtains an alternative expression of the far field intensity at a particular probe position in terms of phase space (Wigner) functions

$$I\left(\mathbf{r}_i,\mathbf{k}_p\right) = \int \mathrm{d}^2r\mathrm{d}^2k\, T_W(\mathbf{r},\mathbf{k})W_i(\mathbf{r}-\mathbf{r}_i,\mathbf{k}_p-\mathbf{k}) . \tag{6}$$

Consequently, the complete ptychographic dataset

$$I\left(\mathbf{r},\mathbf{k}\right) = T_W(\mathbf{r},\mathbf{k}) * W_i(-\mathbf{r},\mathbf{k}) \tag{7}$$

corresponds to a convolution of the mutual transparencies and probe's Wigner transforms in phase space. The mutual object transparency can then be retrieved by suitable deconvolution procedures provided that the phase space

distribution (Wigner function) of the STEM probe is known. The latter has to be determined by suitable reference measurements.

We now set out to relate the mutual object transparency to the optical properties of the studied sample, namely the non-local dielectric function. Follow the derivation of Kohl and Rose [2], we first invoke the notion of the mixed dynamic form factor defined through

$$S(\mathbf{k},\mathbf{k}',\omega) \equiv 2\pi \sum_n \langle E_0 |\hat{\rho}_{\mathbf{k}}| E_n\rangle \langle E_n |\hat{\rho}_{-\mathbf{k}'}| E_0\rangle \,\delta\left(\omega - \frac{1}{\hbar}(E_n - E_0)\right) \quad (8)$$
$$= \int \langle E_0 |\hat{\rho}_{\mathbf{k}}(t)\,\hat{\rho}_{-\mathbf{k}'}(0)| E_0\rangle\, e^{i\omega t}\mathrm{d}t\,,$$

with $|E_n\rangle$ denoting energy eigenstates and $\hat{\rho}_{\mathbf{k}}$ the Fourier transform of the density operator of the many-body electronic system

$$\hat{\rho}_{\mathbf{k}}(t) = \int \sum_n \delta^3(\hat{\mathbf{r}}_n(t) - \mathbf{r}) e^{-i\mathbf{k}\mathbf{r}}\mathrm{d}^3 r \quad (9)$$
$$= \sum_n e^{-i\mathbf{k}\hat{\mathbf{r}}_n(t)}$$
$$= e^{\frac{i}{\hbar}\hat{H}t}\hat{\rho}_{\mathbf{k}} e^{-\frac{i}{\hbar}\hat{H}t}\,.$$

The mixed dynamic form factor is proportional to the transition rate from some non-degenerated ground state $|E_0\rangle$ to $|E_n\rangle$ stimulated by the impinging electron. It is related to the cross-spectral object transparency [6] through a multiplication with the Coulomb potential in Fourier space, i.e., $v_0(\mathbf{k}) = e^2/\left(\epsilon_0 \mathbf{k}^2\right)$, and a kinetic prefactor C_{kin} depending on the acceleration voltage

$$T(\mathbf{k},\mathbf{k}',\omega) = \frac{C_{\mathrm{kin}}}{\hbar^2} v_0(\mathbf{k})\, v_0(\mathbf{k}')\, S(\mathbf{k},\mathbf{k}',\omega)\,. \quad (10)$$

We can now write the non-local dielectric function in terms of a retarded density-density Green's function G [4]

$$\epsilon^{-1}(\mathbf{k},\mathbf{k}',\omega) = \delta(\mathbf{k}-\mathbf{k}') + \frac{1}{\hbar} v_0(\mathbf{k}') \underbrace{\langle\langle \hat{\rho}_{\mathbf{k}}; \hat{\rho}^+_{\mathbf{k}'}\rangle\rangle_\omega}_{G_{\mathbf{k}\mathbf{k}'}(\omega)} \quad (11)$$
$$= \delta(\mathbf{k}-\mathbf{k}') + \frac{1}{\hbar} v_0(\mathbf{k}') \int dt e^{-i\omega t} \langle\langle \hat{\rho}_{\mathbf{k}}(t); \hat{\rho}^+_{\mathbf{k}'}(0)\rangle\rangle$$
$$= \delta(\mathbf{k}-\mathbf{k}') - \frac{i}{\hbar} v_0(\mathbf{k}') \int dt e^{-i\omega t}\Theta(t) \left\langle \left[\hat{\rho}_{\mathbf{k}}(t), \hat{\rho}^+_{\mathbf{k}'}(0)\right]\right\rangle\,,$$

where $[\cdot,\cdot]$ denotes the commutator and $\langle\cdot\rangle$ is an abbreviation for the expectation value over the ground state. By employing the integral representation of the step function

$$\Theta(t) = \frac{1}{2\pi i} \int \frac{e^{i\omega' t}}{\omega' - i\varepsilon} d\omega' \quad (12)$$

and the following representation of the delta function (with $\mathcal{P}$ denoting the Cauchy principal value)

$$\frac{1}{\omega' - i\varepsilon} = i\pi\delta(\omega') + \mathcal{P}\frac{1}{\omega'} \quad (13)$$

we can now reformulate the dielectric function in terms of the mixed dynamic form factor

$$\epsilon^{-1}(\mathbf{k},\mathbf{k}',\omega) = \delta(\mathbf{k}-\mathbf{k}') - \frac{1}{2\pi\hbar} v_0(\mathbf{k}') \int dt d\omega' e^{-i\omega t} \frac{e^{i\omega' t}}{\omega' - i\varepsilon} \left\langle \hat{\rho}_{\mathbf{k}}(t)\hat{\rho}^+_{\mathbf{k}'}(0) - \hat{\rho}^+_{\mathbf{k}'}(0)\hat{\rho}_{\mathbf{k}}(t)\right\rangle \quad (14)$$
$$= \delta(\mathbf{k}-\mathbf{k}') - \frac{1}{2\pi\hbar} v_0(\mathbf{k}') \int d\omega' \frac{S(\mathbf{k},\mathbf{k}',\omega'-\omega) + S(-\mathbf{k}',-\mathbf{k},\omega-\omega')}{\omega' - i\varepsilon}$$
$$= \delta(\mathbf{k}-\mathbf{k}') - \frac{1}{\pi\hbar} v_0(\mathbf{k}') \int d\omega' \frac{S(\mathbf{k},\mathbf{k}',\omega'-\omega)}{\omega' - i\varepsilon}$$
$$= \delta(\mathbf{k}-\mathbf{k}') - \frac{1}{\pi\hbar} v_0(\mathbf{k}')\,\mathcal{P} \int d\omega' \frac{S(\mathbf{k},\mathbf{k}',\omega'-\omega)}{\omega'} + \frac{i}{\hbar} v_0(\mathbf{k}')\, S(\mathbf{k},\mathbf{k}',-\omega)\,,$$

where we made use of $S(\mathbf{k},\mathbf{k}',\omega)=S(-\mathbf{k}',-\mathbf{k},-\omega)$.This expression provides the link between the full EFDP and the non-local dielectric response of the sample. The non-local dielectric function completely describes the optical properties of the (plasmonic) nanostructures, e.g., including dispersion relations. A full reconstruction and analysis of the non-local dielectric response is, however, beyond the scope of this paper. Here we just note that mutual object transparency for inelastic scattering is composed of the product of the elastic and inelastic one within the axial scattering approximation, i.e.,

$$T(\mathbf{r},\mathbf{r}',\omega)=T_{\mathrm{el}}(\mathbf{r},\mathbf{r}',\omega)\,T_{\mathrm{inel}}(\mathbf{r},\mathbf{r}',\omega)\ . \tag{15}$$

Consequently, the elastic contribution, including, e.g., elastic vignetting may be separated from the inelastic one by dividing the total object transparency by the elastic one, which can be reconstructed from an elastic reference measurement.

Supplementary Note 2. Semiclassical Formalism

In the following lines we shortly sketch the derivation of Eq. (2) and Eq. (4) in the main text (employing the same notation), describing the relation between the energy filtered intensity / center of mass and the induced transient fields. We start with noting the time derivative of the expectation of the (time-dependent) Hamiltonian of the beam electron

$$\begin{aligned}\frac{d}{dt}\langle\hat{H}\rangle &= \frac{1}{i\hbar}\langle[\hat{H},\hat{H}]\rangle+\left\langle\frac{\partial\hat{H}}{\partial t}\right\rangle \\ &= -e\left\langle\frac{\partial\Phi^{\mathrm{ind}}(t)}{\partial t}\right\rangle \\ &= -e\boldsymbol{v}(t)\,\boldsymbol{E}^{\mathrm{ind}}(\boldsymbol{r}_e(t),t)\ .\end{aligned} \tag{16}$$

In the last line we assumed that the electron is beam was focused very sharply (to length scales much smaller than the variation of the induced electrical field $\boldsymbol{E}^{\mathrm{ind}}$ or potential Φ^{ind}), so that the density operator of the beam electron may be approximated as a δ-function at the beam position $\boldsymbol{r}_e(t)$. Moreover, we assumed that the strong localization of the beam remained invariant throughout the sample. This approximation is equivalent to that employed for relating the elastic differential phase contrast (DPC) to projected electric or magnetic fields [3] and will be again used further below to express the inelastic IMT in terms of projected fields. The expectation value of the energy loss, which quantum mechanically corresponds to an integral of all possible losses multiplied with the loss probability Γ, may then be written as a classical line integral

$$\begin{aligned}\langle\Delta E(\boldsymbol{r}_{0\perp})\rangle &= \int_0^\infty d\omega\Gamma(\boldsymbol{r}_{0\perp},\omega)\,\hbar\omega \\ &= -e\int_{-\infty}^{\infty} dt\boldsymbol{v}(t)\,\boldsymbol{E}^{\mathrm{ind}}(\boldsymbol{r}_e(t),t)\ .\end{aligned} \tag{17}$$

In the next step we substitute the time with the beam path along z (*non-recoil approximation*) and introduce the spectral representation of the induced electric field

$$\begin{aligned}\langle\Delta E(\boldsymbol{r}_{0\perp})\rangle &= -\frac{e}{\pi}\int_0^\infty d\omega\int_{-\infty}^{\infty} dt\Re\left\{e^{-i\omega t}\boldsymbol{v}\tilde{\boldsymbol{E}}^{\mathrm{ind}}(\boldsymbol{r}_{0\perp}+\boldsymbol{v}(t)\,t,\omega)\right\} \\ &\overset{\mathrm{NRA}}{=} -\frac{e}{\pi}\int_0^\infty d\omega\int_{-\infty}^{\infty} dz\Re\left\{e^{-i\omega z/v_z}\tilde{E}_z^{\mathrm{ind}}(\boldsymbol{r}_{0\perp},z,\omega)\right\}\ .\end{aligned} \tag{18}$$

The experimentally measured spectral density (i.e., energy filtered intensity) and the induced electric field along the optical axis may now be related as follows

$$\Gamma(\boldsymbol{r}_{0\perp},\omega)=-\frac{e}{\pi\hbar\omega}\int_{-\infty}^{\infty} dz\Re\left\{e^{-i\omega z/v_z}\tilde{E}_z^{\mathrm{ind}}(\boldsymbol{r}_{0\perp},z,\omega)\right\}\ . \tag{19}$$

Turning now to the expectation value of the center of mass, we again begin with writing the general time evolution of the lateral kinetic momentum operator

$$\begin{aligned}\frac{d}{dt}\langle\hat{\boldsymbol{p}}_\perp^{\mathrm{can}}+e\boldsymbol{A}_\perp\rangle&=\frac{1}{i\hbar}\langle[\hat{\boldsymbol{p}}_\perp^{\mathrm{can}}+e\boldsymbol{A}_\perp,H]\rangle+\left\langle\frac{\partial\left(\hat{\boldsymbol{p}}_\perp^{\mathrm{can}}+e\boldsymbol{A}_\perp\right)}{\partial t}\right\rangle\\&=-e\left(\boldsymbol{E}_\perp^{\mathrm{ind}}\left(\boldsymbol{r}_e\left(t\right),t\right)+\left[\boldsymbol{v}\times\boldsymbol{B}^{\mathrm{ind}}\left(\boldsymbol{r}_e\left(t\right),t\right)\right]_\perp\right)\ ,\end{aligned}\tag{20}$$

which corresponds to the fundamental Ehrenfest theorem. Again, we assumed for the second line that our beam is focused to a narrow STEM probe. The spectral density of the integrated probability current (i.e., center of mass in diffraction space) can now be represented by a classical line integral, very similar to the energy loss noted previously

$$\begin{aligned}\left\langle\boldsymbol{p}_\perp^{\mathrm{kin}}\left(\boldsymbol{r}_{0\perp}\right)\right\rangle&=\int_0^\infty d\omega\hbar\int I\left(\boldsymbol{k}_\perp,\omega\right)\boldsymbol{k}_\perp d^2k\\&=\int_0^\infty d\omega\Gamma\left(\omega\right)\underbrace{\frac{\int\boldsymbol{j}_\perp\left(\boldsymbol{r}_{0\perp},\omega,\boldsymbol{r}_\perp\right)d^2r_\perp}{\Gamma\left(\omega\right)}}_{\boldsymbol{p}_\perp^{\mathrm{kin}}\left(\boldsymbol{r}_{0\perp},\omega\right)}\\&=-e\int_{-\infty}^\infty dt\boldsymbol{E}_\perp^{\mathrm{ind}}\left(\boldsymbol{r}_e\left(t\right),t\right)+\left[\boldsymbol{v}\times\boldsymbol{B}^{\mathrm{ind}}\left(\boldsymbol{r}_e\left(t\right),t\right)\right]_\perp\ .\end{aligned}\tag{21}$$

Here, I denotes the intensity and $\boldsymbol{j}_\perp$ the lateral current density. Note the normalization with the total energy filtered intensity in the second line. Employing the same transformation steps as above, the momentum transfer reads

$$\begin{aligned}\left\langle\boldsymbol{p}_\perp^{\mathrm{kin}}\left(\boldsymbol{r}_{0\perp}\right)\right\rangle&=-\frac{e}{\pi}\int_0^\infty d\omega\int_{-\infty}^\infty dt\Re\left\{e^{-i\omega t}\left(\tilde{\boldsymbol{E}}_\perp^{\mathrm{ind}}\left(\boldsymbol{r}_e\left(t\right),\omega\right)+\left[\boldsymbol{v}\times\tilde{\boldsymbol{B}}^{\mathrm{ind}}\left(\boldsymbol{r}_e\left(t\right),\omega\right)\right]_\perp\right)\right\}\\&=-\frac{e}{\pi v_z}\int_0^\infty d\omega\int_{-\infty}^\infty dz\Re\left\{e^{-i\omega z/v_z}\left(\tilde{\boldsymbol{E}}_\perp^{\mathrm{ind}}\left(\boldsymbol{r}_{0\perp},z,\omega\right)+v_z\left[-\tilde{B}_y^{\mathrm{ind}},\tilde{B}_x^{\mathrm{ind}}\right]^T\left(\boldsymbol{r}_{0\perp},z,\omega\right)\right)\right\}\ ,\end{aligned}\tag{22}$$

yielding the following relationship between the inelastic momentum transfer (IMT) and the induced transverse fields

$$\boldsymbol{p}_\perp^{\mathrm{kin}}\left(\boldsymbol{r}_{0\perp},\omega\right)=-\frac{e}{\pi v_z}\int_{-\infty}^\infty dz\Re\left\{\frac{e^{-i\omega z/v_z}}{\Gamma\left(\boldsymbol{r}_{0\perp},\omega\right)}\left(\tilde{\boldsymbol{E}}_\perp^{\mathrm{ind}}\left(\boldsymbol{r}_{0\perp},z,\omega\right)+v_z\left[-\tilde{B}_y^{\mathrm{ind}},\tilde{B}_x^{\mathrm{ind}}\right]^T\left(\boldsymbol{r}_{0\perp},z,\omega\right)\right)\right\}\ .\tag{23}$$

Finally, we may replace the induced electric field by the dyadic Green's function and the dielectric susceptibility in the above expression:

$$\begin{aligned}\tilde{\boldsymbol{E}}_\perp^{\mathrm{ind}}\left(\boldsymbol{r}_{0\perp},z,\omega\right)&=-4\pi i\omega\int d^3r'\left[\boldsymbol{G}\left(\boldsymbol{r},\boldsymbol{r}',\omega\right)\boldsymbol{j}\left(\boldsymbol{r}',\omega\right)\right]_\perp\\&=\int d^3r'\left[\boldsymbol{\chi}\left(\boldsymbol{r}_{0\perp},z,\boldsymbol{r}',\omega\right)\tilde{\boldsymbol{E}}^{\mathrm{ext}}\left(\boldsymbol{r}',\omega\right)\right]_\perp\ .\end{aligned}\tag{24}$$

Similar relations, e.g., through the diamagnetic susceptibility, hold for the induced magnetic field.

Supplementary Note 3. Vignetting Effect

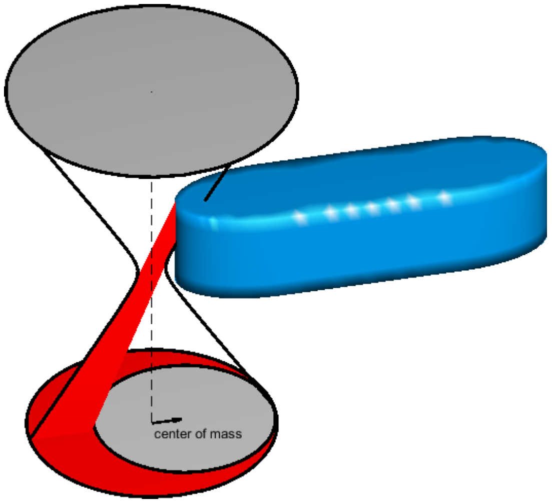

Supplementary Figure 1. Schematic illustration of vignetting effect. Accordingly, the partial obstruction of the convergent illumination on the particle edge leads to a shift of the center of mass (i.e., beam propagation direction) and hence the lateral momentum.

The elastic and inelastic vignetting effect superimposing on the IMT measurement originates from the convergent illumination as illustrated in Fig. 1. Accordingly, the lateral momentum distribution in a convergent electron probe depends on the spatial coordinate. Consequently, an aperture in position space, such as a partial obstruction by the object, may alter the momentum distribution, which is referred to as vignetting. This obstruction may be due to elastic or inelastic scattering. Indeed part of the electrons are elastically scattered into angles larger than the numerical aperture of our optical system. This introduces a partial obstruction of the diffraction disk, referred to as elastic vignetting effect, and hence an apparent momentum shift, which depends on the exact convergence angle of the illumination as illustrated in Fig. 1. Similarly, an inelastic vignetting in the same direction as the elastic one may be introduced, because the probability for exciting a surface plasmon sharply drops for electrons passing inside of the specimen. Note that the details of the modulation of the diffraction disk due to vignetting are involved if taking into account the (partial) coherent nature of the illumination. A comprehensive theoretical description of the latter is beyond the scope of this work.

Supplementary Note 4. IMT Measurements on Gold Nanospheres

Gold is one of the most prevalent material for plasmonic nanostructures due its low dampening and inert surface. In electron microscopy however, gold has the disadvantage of very high scattering absorption: For small collection angles the elastic mean free-path length is about 16 nm, meaning that specimen of suitable thickness (for plasmonics) are essentially intransparent in the TEM. This amplifies the elastic vignetting effect (see Supplementary Note 3) considerably; which is why we deliberately picked aluminum with a much longer mean free-path length of about 100 nm to demonstrate the IMT method in this work. Moreover, Al nanostructures recently became more popular for plasmonic applications, in particular in the UV range, but also because of economical reasons (e.g., [1]).

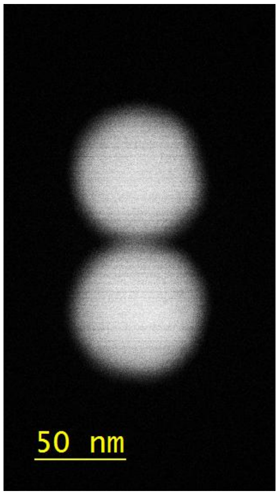

Supplementary Figure 2. High-angle annular dark-field image of a dimer chain of gold nanospheres.

Notwithstanding, we measured the inelastic momentum transfer at chains of gold nanospheres, in particular dimers and trimers of 70 nmspheres (see 2) supported by a carbon foil. The inelastic EFDP of the gold dimer was acquired with a slit centered at 2.5eV (filtering a range from 2eV to 3eV), capturing the most prominent surface transverse mode (Fig. 3), which is homogeneously distributed over the surface. Fig. 4(a) shows the integrated EFDP signal.

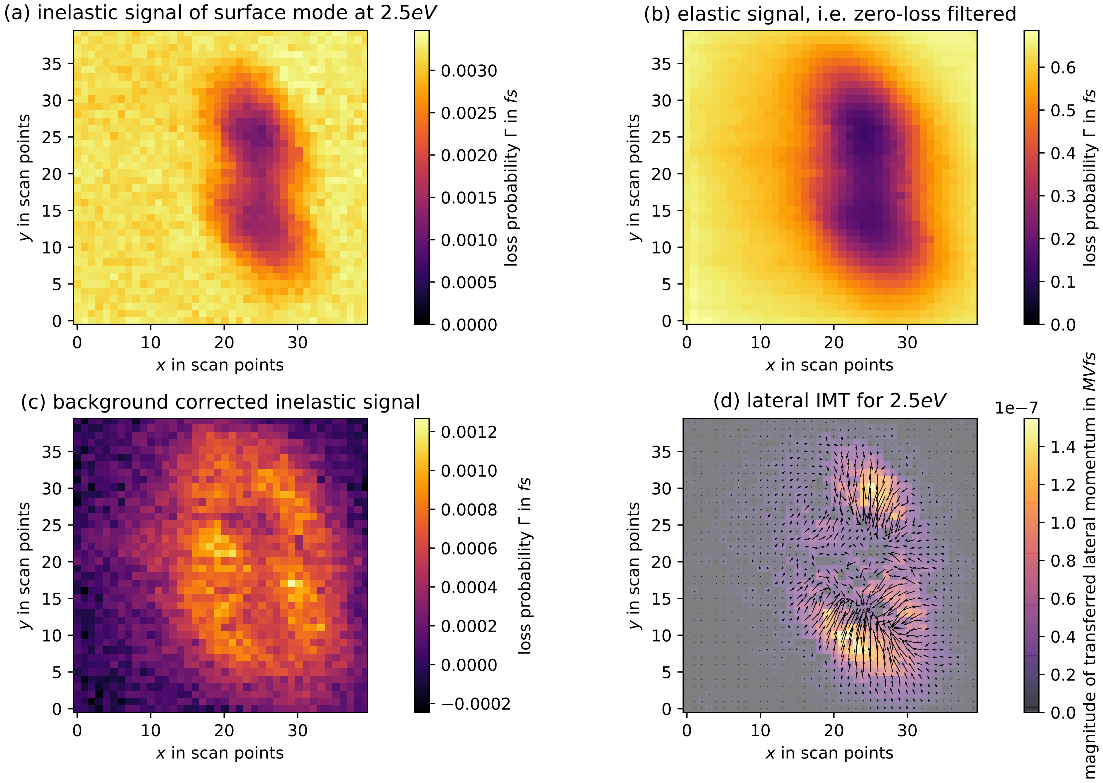

Supplementary Figure 3. Electron energy loss and IMT signals of a gold dimer.

Accordingly, we observe a large constant background from the zero-loss beak and a faint halo from the surface mode. Moreover, a strong damping of the signal on the nanoparticles (much stronger than in Al) is observed, which is clearly of elastic origin as demonstrated by the elastic reference signal 4(b). To remove these effects we subtracted the elastic reference signal (the normalization factor between inelastic and elastic signal has been determined in a region far away from the nanoparticle, in our case the left edge). The resulting background corrected inelastic signal in 3(c) now

clearly shows the halo from the surface plasmon mode. In spite of the low signal-to-noise ratio in that measurement the observed experimental distribution of the loss probability corresponds approximately to the simulated one, where elastic scattering absorption has been taken into account (Fig. 4(a)). Similar to the Al rods the simulated values are approximately one order of magnitude larger than the experimental ones, which we also attribute to the role of the dielectric surrounding (i.e., the substrate and organic ligands). Turning to the evaluation of the lateral momentum

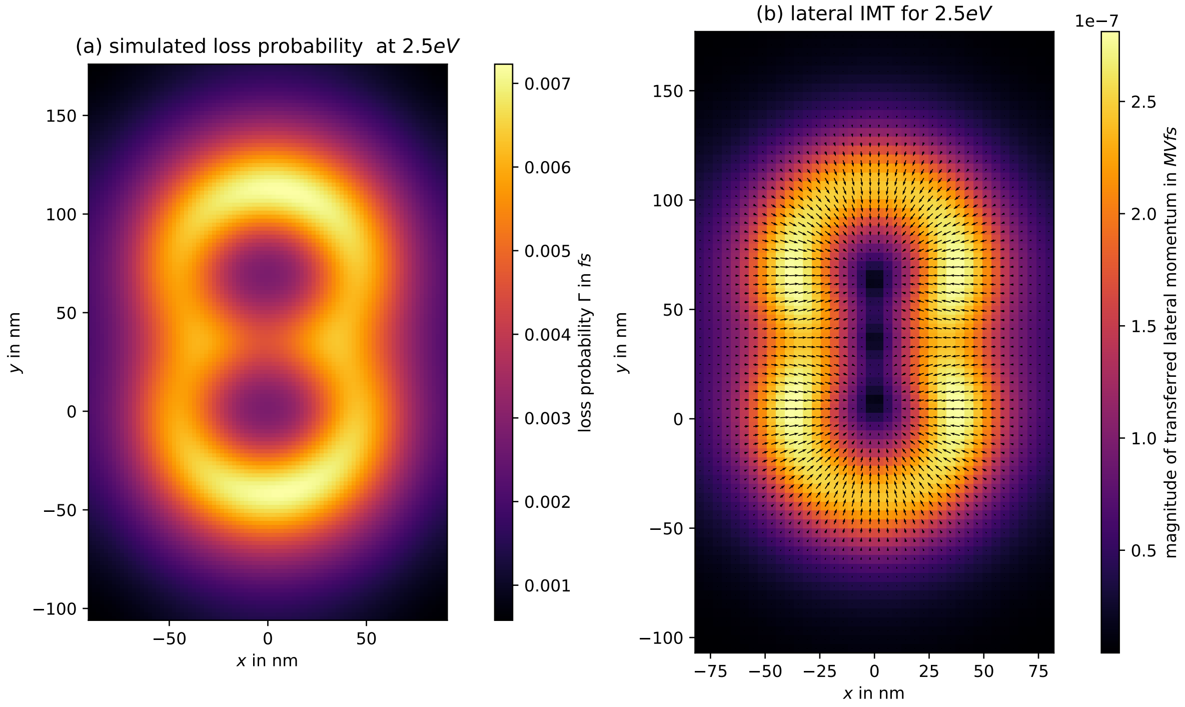

Supplementary Figure 4. Simulated loss signal and IMT for a dimer of gold nanospheres at an energy loss of 2.5eV.

transfer the picture gets more complicated. Similar to the Al case we observe an agreement between the magnitude of the experimental and simulated IMT. However, in the experimental data 3(d) a significant deflection only appears at the ends of the chain and not all around the structure, as expected from the simulation 4(b). We attribute this to the rather strong astigmatism (induced by the monochromator) in the STEM probe, which we could not prevent or compensate during the long acquisition times in the low-mag mode required for the Au sample. This astigmatism stretched the source approximately in horizontal direction, where almost no IMT can be discerned. Noting that the probe momentum distribution is larger along the short axis of the STEM probe in position space and that deflection values of the elastic (not shown) and inelastic signal are about equal, we conclude that elastic vignetting cannot be excluded to be the dominant contributor in the inelastic IMT signal of the Au dimer. In order to remove the elastic vignetting from the IMT signal of samples of heavy elements such as Au one can employ inelastic ptychography (see Eq. 15), which is, however, beyond the scope of this work. Consequently, we do not elaborate further on the significance of the IMT signal obtained from the Au spheres in terms of induced electric fields.

Supplementary References

[1] Mark W. Knight, Nicholas S. King, Lifei Liu, Henry O. Everitt, Peter Nordlander, and Naomi J. Halas. Aluminum for plasmonics. *ACS Nano*, 8(1):834–840, 2014.

[2] H. Kohl and H. Rose. Theory of image formation by inelastically scattered electrons in the electron microscope. *Adv. Electron. Electron. Phys.*, 65:173–227, 1985.

[3] A. Lubk and J. Zweck. Differential phase contrast: An integral perspective. *Phys. Rev. A*, 91:023805, Feb 2015.

[4] W. Nolting. *Vielteilchentheorie*. Springer, Berlin, 2005.

[5] J. M. Rodenburg and R. H. T. Bates. The theory of super-resolution electron microscopy via wigner-distribution deconvolution. *Philosophical Transactions of the Royal Society of London. Series A: Physical and Engineering Sciences*, 339(1655):521–553, 1992.

[6] H. Rose. Image formation by inelastically scattered electrons in electron microscopy. i. *Optik*, 45:139–158, 1976.
